# *Striga* parasitizes transgenic hairy roots of *Zea mays* and provides a tool for studying plant-plant interactions

**DOI:** 10.1186/1746-4811-8-20

**Published:** 2012-06-21

**Authors:** Steven Runo, Sarah Macharia, Amos Alakonya, Jesse Machuka, Neelima Sinha, Julie Scholes

**Affiliations:** 1 Biochemistry and Biotechnology Department, Kenyatta University, P. O. Box 43844, 00100 GPO, Nairobi, Kenya; 2 Division of Plant Biology, University of California Davis, Davis, 1 Shields Avenue LSA 2231, 95616, Davis, CA, USA; 3 Department of Animal and Plant Sciences, University of Sheffield, Sheffield, S10 2TN, United Kingdom

**Keywords:** Maize, *Striga hermonthica*, *Agrobacterium rhizogenes*, Hairy roots, Composite plants

## Abstract

**Background:**

*Striga species* are noxious root hemi-parasitic weeds that debilitate cereal production in sub-Saharan Africa (SSA). Control options for *Striga* are limited and developing *Striga* resistant crop germplasm is regarded as the best and most sustainable control measure. Efforts to improve germplasm for *Striga* resistance by a non-Genetic Modification (GM) approach, for example by exploiting natural resistance, or by a GM approach are constrained by limited information on the biological processes underpinning host-parasite associations. Additionaly, a GM approach is stymied by lack of availability of candidate resistance genes for introduction into hosts and robust transformation methods to validate gene functions. Indeed, a majority of *Striga* hosts, the world’s most cultivated cereals, are recalcitrant to genetic transformation. In maize, the existing protocols for transformation and regeneration are tedious, lengthy, and highly genotype-specific with low efficiency of transformation.

**Results:**

We used *Agrobacterium rhizogenes* strain K599 carrying a reporter gene construct, Green Fluorescent Protein (GFP), to generate transgenic composite maize plants that were challenged with the parasitic plant *Striga hermonthica*. Eighty five percent of maize plants produced transgenic hairy roots expressing GFP. Consistent with most hairy roots produced in other species, transformed maize roots exhibited a hairy root phenotype, the hallmark of *A. rhizogenes* mediated transformation. Transgenic hairy roots resulting from *A. rhizogenes* transformation were readily infected by *S. hermonthica*. There were no significant differences in the number and size of *S. hermonthica* individuals recovered from either transgenic or wild type roots.

**Conclusions:**

This rapid, high throughput, transformation technique will advance our understanding of gene function in parasitic plant-host interactions.

## Background

Parasitic plants are found in 13 angiosperm families and occupy a wide range of habitats. The most economically important parasitic plants are *Striga* and *Orobanche* species of the Orobanchaceae, a monophyletic group of root parasites with approximately 90 genera and more than 2000 species [1]. The *Striga* genus is composed of 30–35 species, over 80% of which are found in Africa, while the rest occur in Asia and the United States. Among the five major *Striga* species, *S. hermonthica* (Del.) Benth. and *S. asiatica* Kuntze. are the most important cereal weeds, whereas *S. gesnerioides* (Willd.) Vatke parasitizes cowpea and other legumes and is a serious constraint to legume production.

The *Striga* life cycle is highly synchronized with that of the host and generally involves the stages of germination, attachment to host, haustorial formation, penetration, establishment of vascular connections, accumulation of nutrients, flowering and seed production [2]. Germination of *Striga* seeds only take place in response to chemical cues, most commonly strigolactones, produced by the host and in some cases non host species [3,4]. It is believed that host-derived chemical signals further guide haustorial formation and subsequent attachment to the host. After penetration of the cortex, haustorial cells undergo a remarkable differentiation process to form vessels that form a continuous bridge with the host xylem [5] that serve as a conduit for host derived nutrients and water.

Economic losses due to *Striga* are enormous. All of the cultivated food-crop cereals (maize, sorghum, millets, wheat and upland rice) are parasitized by one or more *Striga* spp [6]. Overall, *Striga* infests two-thirds of the arable land of Africa and constitutes the biggest single biological cause of crop damage in Africa in terms of grain yield loss, estimated at 40% and worth $US 7 billion annually [7].

Control options for *Striga* are limited. These have generally included modified/improved cultural practices (e.g., crop rotation, intercropping/trap crops, different planting techniques, hand weeding, management of soil fertility), use of herbicide containing seed dressing, direct chemical treatment of soil to reduce seed levels in the soil, and identification of resistant (the ability of a host to prevent/limit *Striga* attachment/growth) and/or tolerant (the ability of a host to maintain biomass and yield in spite of *Striga* infection) germplasm for directed breeding [6].

Overall, *Striga* management practices are limited by our understanding of the biology of the parasite-host interaction. Such information is vital for development of appropriate management strategies using both genetic modification (GM) and non-GM approaches [8]. With the ongoing parasitic plant genome project (http://ppgp.huck.psu.edu/), parasitic plants are fast entering the genomics era. These efforts will bring to light a large number of genes (including resistance genes) with unknown functions, underscoring the need for functional genomics tools for studying host-parasite interactions [9].

We hypothesized that many genes involved in *Striga*-host interactions are expressed in roots, thus a genetic transformation method that rapidly and efficiently generates a large number of transgenic host roots would provide an excellent system for studying the functions of genes involved in all aspects of *Striga*-host interactions.

The soil bacterium *Agrobacterium rhizogenes* is a naturally occurring plant pathogen [10] that can transfer T-DNA into the genomic DNA of plants. Infected plant cells that integrate a root inducing (Ri) plasmid-derived T-DNA from *A. rhizogenes* develop a large number of neoplastic, plagiotropic transformed ‘hairy’ roots [11]. The feasibility of using *A. rhizogenes* in plant transformation has been demonstrated in a diverse array of plant families [11-15] for various applications e.g. production of stably transformed plants, [16,17], gene analysis, [18-20] secondary metabolite production reviewed in [21], plant-microbe interactions [18] and plant-pathogen interactions [22].

Of the diverse range of *A. rhizogenes* mediated transformation applications, a key milestone was the development of ‘composite’ plants [23]. The term ‘composite’ plant was coined to describe plants that have a wild type shoot and a transformed root stock. Composite plants present an ideal system for gene function studies of plants in association with other organisms. As such, they have been extensively used in analyses involving infection of legumes with rhizobia and nitrogen fixation [24,25] as well as host plant associations with mycorrhiza [24]. In general, composite plants offer the following advantages; (i) root biology can be studied in the roots of whole plants rather than in axenic cultures, (ii) since every transformed root is an individual event, multiple transgenic events can be obtained in a single transformation experiment, and (iii) they can be maintained outside of tissue culture after induction [26] so the amount of time required to generate transgenic plant tissue in transformation is greatly reduced.

Despite successful application of composite plants in elucidating plant-microbe interactions, the importance of maize as a model for genetics, the importance of *Striga* as a root parasite, and the enormous amount of host-parasite interaction data obtainable from composite hairy roots, no transgenic hairy root composite system has been developed for any of the *Striga* hosts. Here we show that *A. rhizogenes* can be used to efficiently produce transgenic hairy roots in maize. We further show that transgenic roots of composite maize plants can be infected by the parasitic plant *S. hermonthica* and that this system can be used to study *Striga*-maize interactions as a functional genomics tool.

## Results

### *Agrobacterium rhizogenes* induces hairy roots in maize producing composite plants

*Agrobacterium rhizogenes* composite plants are generally produced from wounding plant tissue e.g. leaves, cotyledons, or root hypocotyls, followed by inoculation with a culture of bacteria under *in vitro* or *in vivo* conditions. We used a protocol that combined both *in vitro* and *in vivo* procedures. In summary maize composite plants were produced as follows: 5 day old maize seedlings were infected with *A. rhizogenes* K599 harbouring a GFP reporter construct by an excision that split the main root in two halves approximately 4 mm from the tip. Later, seedlings were co-cultivated with bacteria for three days and left on hormone free MS resting media for 10 days to allow hairy root development.

**Figure 1:**
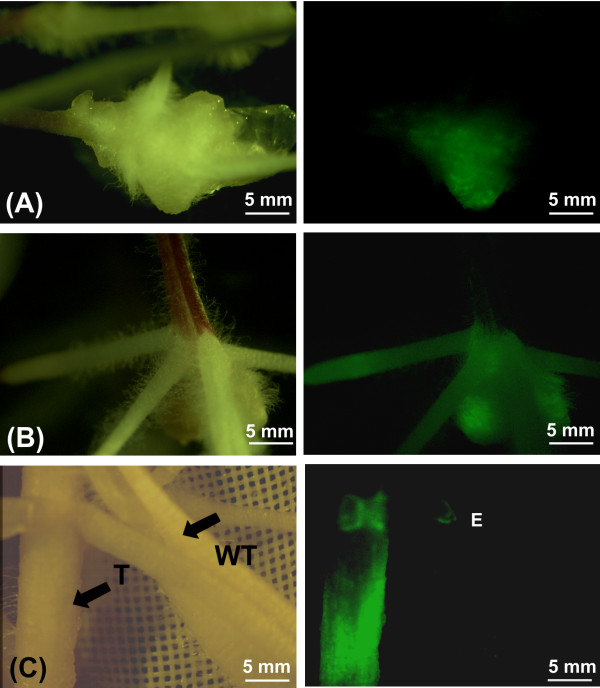
**The morphology of maize roots transformed with*Agrobacterium rhizogenes*strain K599 harbouring a GFP reporter gene construct.** Figures **A** and **B** show transformed maize roots in phytotrays and **C** shows a transformed root in a rhizotron under bright field (left column) and UV irradiance (right column). (**A**) Tumours that formed 10 days after infection with *A. rhizogenes,* from which hairy roots emerge. (**B**) Highly transgenic hairy roots that formed from wounded sites (15 days after inoculation with *A. rhizogenes*). (**C**) Transgenic roots that sometimes formed along with wild type roots. Composite plants consisted of a mosaic of transformed roots that expressed GFP (T) and wild type (WT) from normal maize growth that did not show GFP expression. GFP fluorescence can also be seen on an emerging root at the point labelled ‘E’.

The time course of development of transgenic hairy roots from maize is described in Figure 1. Typically, the root elongated and a globular tumour developed 7-10 days later (Figure 1A). Hairy roots started emerging from the tumour after a further 7 days (Figure 1B). At the end of the third week (21 days) of infection, all plants had developed roots from tumours and were transferred to rhizotrons which are transparent root observation chambers [27]. In rhizotrons, composite plants maintained a normal above ground wild type phenotype while transgenic roots continued to proliferate throughout the rest of their growing cycle (Figure 1C).

In many plant species transgenic roots induced by *A. rhizogenes* are characterized by fast growth, high lateral branching and plagiotropism. We identified two root phenotypes in maize: friable callus which gave rise to roots (Figure 1A) and highly branched and plagiotropic roots (Figure 1B). Whereas highly branched plagiotropic roots were observed throughout the growth of the composite plants, the friable callus phenotype was only observed during the initial stages of hairy root development.

Wounded maize seedlings from the genotype used (CML 216) showed high susceptibility to *A. rhizogenes* strain K599 and efficient uptake of the T-DNA containing the GFP reporter gene. To assess transformation efficiency, composite plants growing in rhizotrons were scored for GFP expression using a charge-coupled device camera (CCD) (Diagnostic Instruments Inc.) mounted on a Leica MZFLIII stereomicroscope (Leica Instruments GmbH). On average, 85.3% ± 16.2 of seedlings infected with *A. rhizogenes* produced at least one transgenic root. The percentage of transgenic roots per composite plant was 38.4% ± 5.6 three weeks after transformation. These values represent an average of 5 plants ± the standard deviation from three independent experiments.

### *Agrobacterium rhizogenes* transferred T-DNA is integrated and expressed in maize tissue

In composite plants, wild type roots continue to appear as part of the normal plant development process independent of *A. rhizogenes* inoculation. As a result, the maize plant root system becomes a mosaic of transformed and wild type roots. To distinguish between these two classes of roots, we used the pMDC44 binary vector (Gateway Technologies Invitrogen, Carlsbad, CA, USA) carrying GFP reporter plasmid.

Transformed roots showed a high intensity of GFP expression that was not present in wild type roots (Figure 1C). Overall the 35 S-driven GFP expression pattern was highest in root tips (Figures 1B and C) or uniformly present in the whole root (Figure 1C). The globular tumours at the site of emergence of new transgenic roots and sites of incipient lateral roots also showed strong GFP fluorescence (Figure 1A and B). Expression of GFP activity confirmed nuclear integration of the transgene in the plant cells.

**Figure 2:**
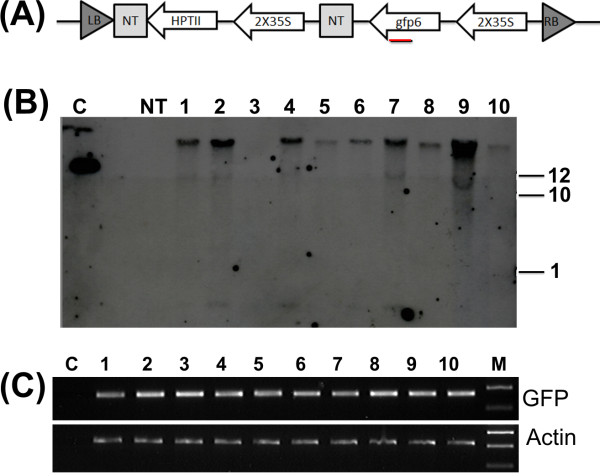
**Detection of transgenic hairy roots by Southern hybridization and Reverse Transcription PCR.** (**A**) Schematic of GFP gene from pMDC44 showing the 35 S promoter, GFP gene, NOS terminator, HPT selection (Gateway Technologies Invitrogen, Carlsbad, CA, USA). Red bold line show the position used to amplify the GFP gene. Presence of GFP results in a 345 bp fragment. (**B**) Southern blot of transgenic roots. Lane 1 C is positive control (0.05 ng of pMDC44 plasmid), Lane 2 no sample loaded, Lane 3 NT non transformed wild type roots, lanes 4-13 consist of roots from 10 composite plants selected randomly (**C**) RT-PCR on transgenic and non-transgenic root cultures. The panel shows results obtained from using GFP specific primers and the same substrates amplified with Actin primers for loading control (lower panel). Lane 1 is 1 Kb ladder (Hyperladder I – Bioline) Lanes 2-11 cDNA *Zea mays* roots of composite plants, lane 12 cDNA from wild type formed maize roots.

In addition to GFP expression, the successful integration and stable expression of the T-DNA containing the GFP reporter gene construct (Figure 2A) in maize roots was confirmed by Southern blotting and reverse transcription-PCR (RT-PCR) (Figure 2B and C). Genomic DNA of roots expressing GFP was digested with *Hind*III which cuts only once within the T-DNA. Restriction-digested DNA was then blotted and hybridized with a 345 bp alkaline phosphatase labelled fragment as a probe. As shown in Figure 2B, the ten randomly selected composite plants showed between 1 and 3 integration events of the *GFP* gene thereby confirming their transgenic nature. No hybridization signal was observed in the control plant.

Ten independent composite plants were randomly selected and the expression levels of GFP were measured by RT-PCR. A maize actin gene (GenBank accession no: AY107106) was used as the reference gene. As expected, the transgenic GFP lines 1–10 expressed GFP, whereas no expression was detected in the control plants (Figure 2C).

### *Striga hermonthica* infects transgenic hairy roots of maize

**Figure 3:**
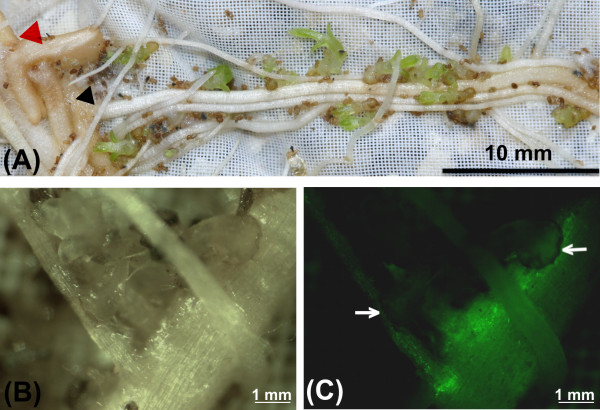
**(A) Roots of a composite maize plant (variety CML 216) growing in a rhizotron 15 days after inoculation with*S. hermonthica.*** The red arrow points to the site of infection with *A. rhizogenes* while the black arrows indicate transgenic roots resulting from infection. (**B** and **C**) show close up photographs of two *S. hermonthica* seedlings infecting transformed maize roots viewed under bright field and UV irradiance respectively. Arrows point to the *S. hermonthica* attachments.

To determine whether the *A. rhizogenes* transformation affected the normal *Striga* infection process, wild type and transgenic hairy roots (selected using GFP fluorescence as a marker) were infected with germinated *S. hermonthica* seeds (Figure 3).

Numerous *S. hermonthica* attachments were visible on wild type and transgenic hairy roots 15 days after infection (Figure 3A). There was no significant difference between the number and size of *S. hermonthica* individuals infecting transformed or wild type roots (*P < 0.05*) (Table 1). These data indicate that *Striga* could initiate and complete its life cycle on hairy roots in exactly the same manner as on wild type roots. Figures 3A and B show close up images of *S. hermonthica* individuals attached to a transgenic root under bright field and under irradiance (Figure 3C).

**Table 1:** **Infection of transformed and wild type roots by*Striga hermonthica***

	**No. of *Striga* plants**	**Length of *Striga*plants (cm)**
Wild type	53.8 ± 10.4	2.5 ± 0.5
Transformed	53.6 ± 9.3	3.0 ± 0.3

Each value is an average of 10 plants ± the standard deviation from 3 independent experiments.

Data for wildtype and transformed plants were not significantly different at *P < 0.05* (ANOVA).

**Figure 4:**
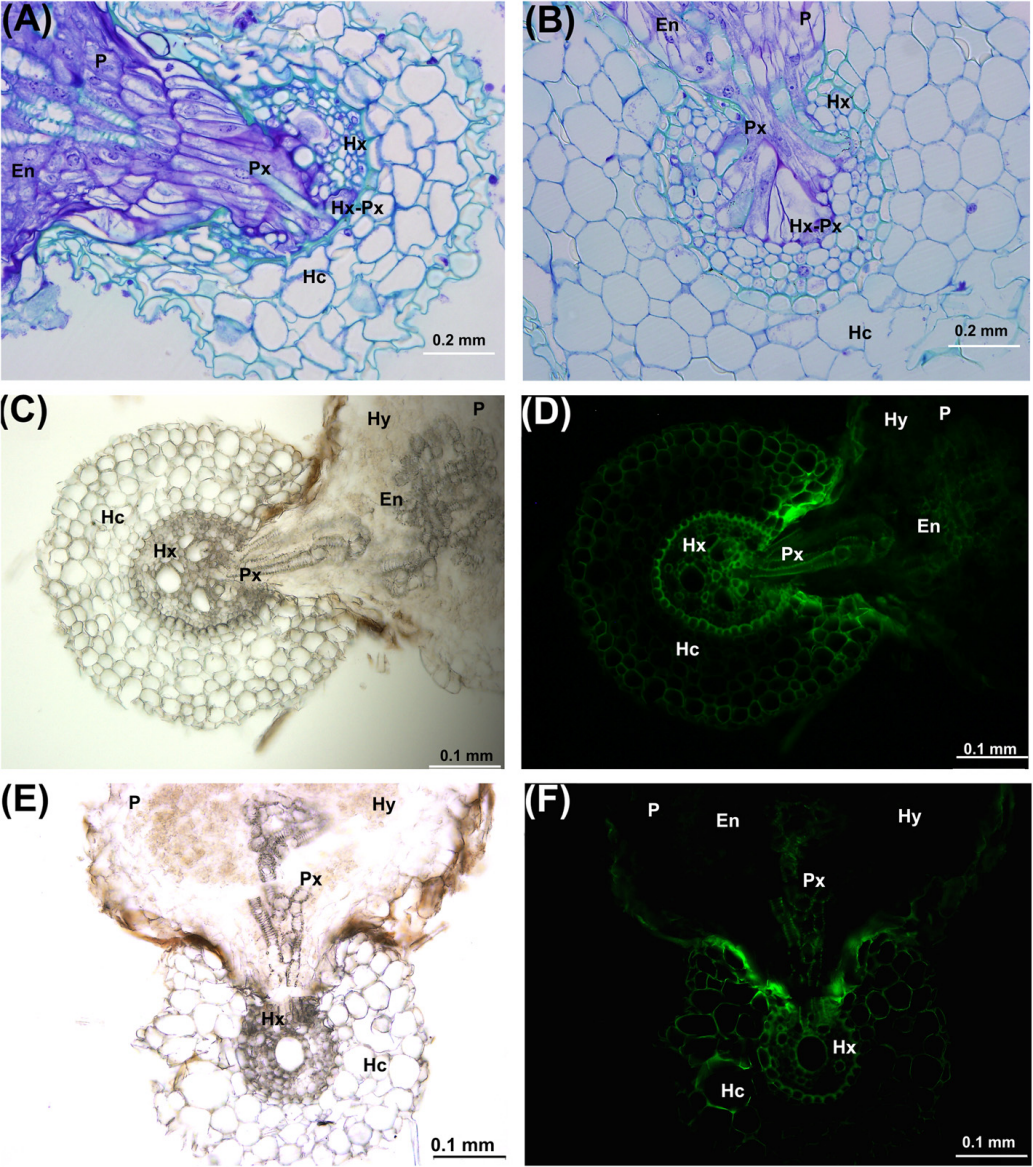
**Transverse sections through roots of maize, 15 days after infection with*S. hermonthica.*** (**A** and **B**) show cross sections of a wild type maize root (**A**) and a transgenic hairy root (**B**) infected with *S. hermonthica* (embedded in Technovit resin). **C** and **D** show a bright field and UV fluorescence image, respectively, of a cross section through a frozen hairy root infected with *S. hermonthica*. The parasite has traversed the root cortex and formed connections with the host xylem vessels. (**E** and **F**) show cross sections of a wild type maize root infected with *S. hermonthica* under bright field and UV irradiance. En, endophyte (internal part of haustorium); Hc, host root cortex; He, host endodermis; Hx, host xylem; Hx–Px, host–parasite xylem continuity; Hy, hyaline body; P, parasite haustorium; and Px, parasite xylem vessels.

To determine if there were morphological differences in the way in which *S. hermonthica* penetrated the transformed compared to wild type maize roots, root samples plus *S. hermonthica* attachments were either frozen (using a freezing microtome) or embedded in methyl methacrylate (Technovit® TAAB, UK), sectioned and viewed using an Olympus BX51 microscope (Figure 4). Figure 4A and B show cross sections of a wild type and transgenic root, respectively, following infection with *S. hermonthica*. In both cases the images show that the parasite has penetrated the root cortex and endodermis and has fused its xylem vessels with those of the host thus establishing a functional continuum between *Striga* and maize roots. There was no difference in the timing or characteristics of the infection process in transgenic compared to wild type roots. Figures 4C and D show a section through a frozen hairy root of maize infected with *S. hermonthica* viewed under bright field and UV irradiance respectively. Again it can be seen that *S. hermonthica* has successfully infected the root. The green fluorescence in the UV illuminated root is largely due to autofluorescence from cell walls as this is also present in infected wild type roots (Figure 4E and 4F). Together these data indicate that the parasitism process was successful in transgenic roots of maize.

## Discussion

We have for the first time, established a transformation protocol that allows the production of transformed maize roots at the *A. rhizogenes* infection site leading to development of composite plants. As a functional genomics tool, the protocol provides three distinct advantages. Firstly, the protocol is highly efficient with 85.3% of inoculated plants producing at least one transgenic root. Hitherto, the highest transformation efficiency achieved in maize roots was 50% [28] using *A. tumefaciens* mediated transformation on immature zygotic embryos. Secondly, the procedure is rapid and not laborious. It resulted in the production of acclimatized composite plants bearing well-developed transformed rootstocks in one month, making it directly usable for rapid validation and functional studies of gene expression in the roots. In comparison, a standard transformation procedure usually takes up to 14 months to produce similar well-developed transformed plantlets starting from primary explants, i.e. immature zygotic embryos. Thirdly a great number of independent transformation events can be obtained and analyzed in a single plant because every transgenic root originates from a single cell [29,30] and represents an independent transformation event.

In the past, maize transformation has been achieved using *A. tumefaciens* and immature zygotic embryos [31,32] leaf discs [33] apices [34,35] and using *A. rhizogenes* on immature zygotic embryos [36]. We combined the efficiency of producing transgenic hairy roots induced by *A. rhizogenes in vitro* with the versatility of root observation chambers termed rhizotrons to develop a composite maize system that can be used to study phenotypes involved in the maize-*Striga* interaction. Our system involved germinating maize, inoculating and co-cultivating with bacteria, incubating in hormone free MS media, all under sterile *in vitro* conditions, followed by transfer to transparent, non sterile, root observation chambers. We showed that *Striga* attaches to transgenic hairy roots of maize, develops haustoria and penetrates the root in a manner identical to that seen in wild type roots demonstrating that composite maize roots retain the host ability for *Striga* parasitism. Combining *in vitro* and *in vivo* procedures for developing composite plants proved attractive because; (i) culture conditions could be optimized to allow efficient generation and proliferation of hairy roots (ii) it provided the unique opportunity to conduct a detailed time course macroscopic and microscopic observation of *Striga*-maize interactions in a soil free environment (rhizotrons), (iii) it avoided the critical step of *Striga* sterilization and the subsequent difficulty in maintaining *in vitro* culture conditions involving maize roots, *Striga*, and *A. rhizogenes* and (iv) allowed studies on whole plants which went through the entire growth cycle.

When *A. rhizogenes* infects plant tissue, it transfers its T-DNA (also termed R-DNA) into the plant genome. The hallmark of this transfer is proliferation of highly branched, plagiotropic, and sometimes thicker than normal roots. We observed two classes of root phenotypes which have also been reported before in [37] either occurring together or individually for different transformation experiments. In all reported cases, the callus phenotype does not persist for long into the plant’s growth cycle. These alterations in root morphology arise from the integration and expression in the plant cell of oncogenes such as the *ROL* genes, *AUX* genes involved in auxin synthesis or genes synthesizing opines, borne by the T-DNA of the Ri plasmid [38].

Despite the changes induced by the transferred R- plasmid from *A. rhizogenes* to maize roots (plagiotropism, lateral branching and root hairs); there were no differences in morphology and development of *Striga* on transgenic roots compared to wild type. Indeed, the cell arrangement in transgenic and wild type roots was identical. At 21 days after infection, a cross-section through the parasite haustorium showed well developed *Striga*-maize hairy root xylem–xylem connections. Furthermore, *Striga* had a clearly differentiated vascular core and hyaline body. This mature haustorium is crucial for successful *Striga* parasitism because; (i) xylem–xylem connections allow the movement of solutes from host to parasite and, (ii) the hyaline body is thought to metabolize these solutes, and further regulate the supply of nutrients to the developing parasite.

In addition to the *ROL* and *AUX* genes, *A. rhizogenes* also transfers the T-DNA, (just like *A. tumefaciens*) of the binary vector when co-transferred [16,39], allowing the integration of a foreign gene. To confirm transfer of T-DNA from *A. rhizogenes* and its expression in plant cells, we mobilized a binary vector containing the *GFP* gene into the hypervirulent *A. rhizogenes* strain K599. Expression of GFP in composite roots allowed for a rapid and efficient visual selection of transgenic roots avoiding the selection of co-transformed roots with antibiotics or herbicides. GFP expression was evident mostly in the vascular cylinder and root tips of transformed hairy roots. In some cases, GFP expression varied non-uniformly probably because of the nature of the promoter used (35 S CAMV) as reported previously [40,41]. Variation in activity could also be due to the copy number of the integrated *GFP* gene copies or chromosomal insertion site (position effect) [42]. In general, intense fluorescence was observed in actively dividing cells such as the root meristem and sites of incipient root hair primordia. Clear GFP expression is indicative of complete and stable integration of the T-DNA gene. Additional evidence for integration of the transgene was provided by Southern blot and RTPCR.

Gene expression studies using GFP and RTPCR demonstrated that *A. rhizogenes*-mediated transformation is a potent tool to produce transformed maize roots using a binary vector. In principal then, maize can be transformed with a binary vector harbouring any gene of interest and this can be used as a rapid method to screen for phenotypes that are expressed in the roots. We envision that in the future this system will be useful in analysis of candidate genes for *Striga* resistance and for validating their function in host-parasite interactions for example in reverse genetic studies. Moreover, the system can be applied in gene discovery, for screening for *Striga* resistant genes or other genes involved in parasitism. By exploiting the hairy root inducing properties of *A. rhizogenes*, it is possible to transform numerous roots and obtain gain of function mutants for genes that are expressed in the roots for example through activation tagging. For example, genes known to have critical roles in *Striga* parasitism or host defense, identified in large genome sequencing projects or fine mapping studies can be cloned into high-throughput vectors [43] and transformed into *A. rhizogenes.* Such constructs can be used for transgene over-expression or RNAi mediated gene suppression. For ease of tracking transformation events, constructs can be fused to a reporter gene such as GFP. For over-expression vectors, the DNA fragment can be cloned downstream of a constitutive promoter, upstream to a GFP sequence fused in-frame. For down-regulation, transformed roots can be tracked by GFP in constructs designed as promoter::DNA sense-DNA antisense-promoter::GFP:terminator.

## Conclusions

Maize is an important food source in SSA and also a good model for studying host-*Striga* interactions. Efforts to improve maize through genetic engineering approaches have been limited because of lack of efficient and rapid protocols for transformation. This work describes an efficient, rapid protocol for the generation of transformed hairy roots in maize using *A. rhizogenes* harbouring a GFP reporter gene in a binary vector. Transformed maize roots expressed GFP and integrated the transgene into their genome. In addition, transformed maize roots retained their susceptibility to *S. hermonthica* and became infected at the same frequency as wild type roots. This technique is suitable for use in functional genomics analyses for genes whose phenotypes are manifested in the roots. This methodology represents a significant advantage over existing transformation protocols which are expensive and time consuming.

## Methods

### Seed sterilization and germination

CML 216 is a subtropical white maize inbred line developed in Africa by plant breeders at the International Maize and Wheat Improvement Centre (CIMMYT). Seeds obtained from CIMMYT (Nairobi) were first surface sterilized in 70% ethanol (5 min) and 10% (v/v) commercial bleach for 15 min then rinsed three times in sterile water. Ten seeds were placed in 90 mm Petri plates on hormone free MS medium [44] containing vitamins and 8% agar (Sigma-Aldrich). Seeds were germinated in a growth room at 25 °C in the dark for 5 days.

### *Agrobacterium* strain and binary vector

*Agrobacterium rhizogenes* strain K599, a cucumopine type was tested for its ability to induce transformed hairy roots in maize seedlings. The binary vector pMDC44 (Gateway Technologies Invitrogen, Carlsbad, CA, USA) was introduced into bacteria by electroporation. The vector has a *GREEN FLUORESCENT PROTEIN* (*GFP*) under control of 35 S cauliflower mosaic virus promoter and a *NOPOLINE SYNTHASE* (*NOS*) terminator (Figure 2A).

To prepare *A. rhizogenes* bacteria for infecting maize seedlings, the protocol developed for soybean transformation [25] was modified and used. Briefly, bacteria harbouring the pMDC44 binary vector were streaked (from a glycerol stock) onto the surface of Luria-Bertani (LB) plates containing Kanamycin 100 μg ml^–1^ and incubated at 28 °C for 2 days. A single colony was re-streaked onto a fresh plate and incubated at 28 °C for 2 days. One loop of fresh bacterial culture from the plate was re-suspended in 1 ml of liquid LB medium containing 15% (v/v) glycerol and 200 ml of the suspension was spread onto the surface of LB plates containing Kanamycin 100 μg ml^–1^ and incubated at 28 °C overnight.

### Production of transgenic hairy roots

Bacteria were collected from the plates by scraping with a scalpel blade and used to inoculate 5 days old maize seedlings. The main root was infected with *A. rhizogenes* by splitting the root tip (4 mm from the base) with a scalpel blade coated with *A. rhizogenes* culture. Seedlings were co-cultivated with bacteria at 28 °C for 3 days by placing the infected roots on MS medium supplemented with acetosyringone (50 μg ml^−1^) and solidified by adding 8 g L^−1^ Agar, in Phytotray II culture containers (Sigma-Aldrich). Co-cultured germinated seedlings were washed in liquid hormone free Murashige and Skoog (MS) medium containing Cefotaxime (500 μg ml^−1^) for 10 min before culturing on hormone free MS media containing Cefotaxime (250 μg ml^−1^) in Phytotray II containers (Sigma-Aldrich).

Plants were maintained on MS media for 10 days then transferred to rhizotrons – 25 cm × 25 cm root observation perspex chambers filled with vermiculite [27]. These systems allowed monitoring of parasite development on hairy roots in a non-destructive manner over time. In addition, rhizotrons provided access to the roots for harvesting parasite and host root material for molecular and histological analyses. Plants were maintained in rhizotrons for 10 days while being drip-fed with 40% Long Ashton solution containing 1 mol per L ammonium nitrate [45] at four intervals during the photoperiod to give a total volume of 200 ml day per day. Plants were grown in a controlled environment, walk-in, growth chamber with a 12-h photoperiod and a photon-flux density of 800 μ mol quanta m^-2^ s^-1^ at plant height. Day night temperatures were maintained at 28°C : 24°C, and relative humidity was maintained at 60%.

The development of hairy roots was observed at different stages; in phytotrays after co-cultivation (after 3 days), during maturation (after 10 days) and in rhizotrons (after 21 days). Images of GFP expression were taken using a CCD camera (Diagnostic Instruments Inc.) mounted on a Leica MZFLIII stereomicroscope (Leica Instruments GmbH).

### Infection of wild type and composite plants with *Striga*

Ten days after transfer into rhizotrons, composite and wild type maize plants had well developed roots. Both wild type and transgenic hairy roots were inoculated with 20 mg of germinated *S. hermonthica* seeds which were aligned along the host roots using a fine paint brush. Germination of the *S. hermonthica* seeds was triggered by the addition of an artificial germination stimulant GR24 (0.1 ppm) 18 hours prior to infection [27]. Germination of *S. hermonthica* seeds prior to infection ensured synchronous attachment to the host roots. After infection with *Striga*, plants were returned to the controlled environment growth room.

To determine if *Striga* had attached to transgenic maize roots, images of GFP expression were taken using a CCD camera (Diagnostic Instruments Inc.) mounted on a Leica MZFLIII stereomicroscope (Leica Instruments GmbH) after 10 and 15 days. *Striga* plants were harvested from the roots of both transformed and wild type roots 21 days after infection. Harvested *Striga* plants from each host plant were placed in a 90 mm Petri plate and photographed. The number and length of *Striga* plants on each host plant was calculated from the photographs using image analysis software (ImagePro, Media Cybernetics). Five replicate plants were used for each treatment (control or transgenic) in three independent experiments. Statistical analysis of data (Analysis of Variance (ANOVA) was performed using Minitab version 15 (Minitab Inc., USA).

### Microscopy

To examine the extent of parasite development within the host root cortex, small sections of wild type and transgenic hairy roots plus *S. hermonthica* attachments were fixed using Carnoys fixative (4 : 1, 100% ethanol : acetic acid) and vacuum infiltrated for 20 min. Samples were then embedded using Technovit 7100 kit (TAAB, UK) as described in [46]. Five micron thick sections were cut using a Leica R12145 microtome (Leica Instruments GmbH) and transferred to microscope slides (poly-lysine slides; SLS, Nottingham, UK) using forceps. Sections were stained with 0.1% toluidine blue O (BDH) for 20 s, washed in distilled water and dried at 65°C for 30 min on a hot plate. Sections were mounted with DePex (BDH, Poole, UK) and observed and photographed using an Olympus BX51 microscope and DP 71 camera (Olympus Optical Ltd, London, UK).

Transgenic hairy roots that showed GFP expression and wild type roots were also embedded in tissue-freezing medium at -20 \\°C in a Leica CM1900 cryostat (Sakura Finetek Tissue*-*Tek® O.C.T^TM^). Frozen sections, 40 μm thick, were cut with a Leica CM1900 cryostat (Leica Instruments GmbH) and mounted in sterile distilled water. Bright field images were taken immediately using an Olympus BX51 microscope and DP 71 camera (Olympus Optical Ltd, London, UK). Images of fluorescence were acquired using a U-MWIBA2 filter cube with the same microscope and camera. A xenon arc lamp together with a 470–490 nm excitation filter provided a 515–550 nm barrier filter or a 330–385 excitation and a 420 nm long pass filter.

### RNA isolation and reverse transcriptase PCR

For total RNA extraction, approximately 100 mg of fresh young leaves were harvested from roots showing GFP expression with the Plant RNeasy mini kit (Qiagen, UK) following the manufacturer’s instructions. After extraction, total RNA preparations were treated with RNase-free DNase (1 unit for 30 min at 37°C; Invitrogen, UK) to ensure the complete removal of genomic DNA. Reverse transcription PCR (RT-PCR) amplification was performed with the SuperScript II First-Strand Synthesis System kit (Invitrogen, UK) according to manufacturer’s instructions with oligo(dT) 12–18 primers. One μg of purified total RNA was used in each RT-PCR amplification.

The *ACTIN and GFP* transcripts were identified by amplifying a 426 bp and 345 bp fragments, respectively, using primer pairs (GFP forward 5′ CCTACGGCGTGCAGTGCTTCAGC 3′ GFP reverse 5′ CGGCGAGCTGCACGCTGCGTCCTC 3′. ZmAct forward 5’ ACCCAA AGGCTAACCGTGAG 3’ ZmAct reverse 5’ TAGTCCAGGGCAATGTAGGC 3’. Twenty-five μl cycle PCR reactions were set up as follows: 2.5 μl of 5 × PCR buffer (Bioline, UK), 10 μM forward and reverse primers, 0.15 μl of 5 u μl^-1^ MyTaq^TM^ DNA polymerase (Bioline, UK); 1.5 μl of cDNA template and 18.35 μl of nuclease free water. The PCR was carried out using the following cycling conditions: 94°C for 3 min – 1 cycle; 94°C for 30 s, 55 °C for 30 s, 72°C for 30 s – 20 cycles; and 72°C for 2 min.

### Detection of GFP in transgenic hairy roots by Southern blot

Only roots that showed GFP expression were excised for DNA extraction. DNA was extracted from homogenized tissue using urea extraction buffer containing 1% (v/v) Sarcoyl (Sigma-Aldrich). Extractions were phenol:chloroform extracted at a 1:1 ratio and incubated at room temperature for 30 min. DNA was ethanol precipitated from the supernatant and treated with 100 U RNase A (Qiagen UK). Samples were re-precipitated with isopropanol and re-suspended in nuclease free water. Approximately 10 μg of DNA from each sample was digested overnight with *Hind*III restriction enzyme. Digested DNA was ethanol precipitated overnight and then separated by electrophoresis in 0.8% agarose gel buffered in 1 × TAE. DNA was transferred to a Hybond-N + (Amersham Biosciences) nylon membrane and cross-linked. A 345 bp fragment of the GFP gene amplified using primer pairs (GFP forward 5′ CCTACGGCGTGCAGTGCTTCAGC 3′ GFP reverse 5′ CGGCGAGCTGCACGCTGCGTCCTC 3′ was labelled and used as a probe. Labelling hybridization and detection were carried out according to the manufacturer’s instructions using the CDP star labelling system (Amersham Biosciences). Wild type maize (not infected with *A. rhizogenes*) was used as a negative control.

## Abbreviations

GFP, Green Fluorescent Protein; GM, Genetic modification; SSA, Sub-Saharan Africa.

## Competing interests

The authors declare that they have no competing interests.

## Author’s contribution

SR performed the experiments and participated in preparation of the manuscript. SM and AA participated in the experiments. JM and NS conceptualized the experiments on maize genetic transformation with *A. rhizogenes* and participated in preparation of the manuscript. JS conceptualized the experiments on infecting transgenic hairy roots with *S. hermonthica* and participated in the preparation of the manuscript. All authors have read and approved the final manuscript.
